# Changes in Visual Object Recognition Precede the Shape Bias in Early Noun Learning

**DOI:** 10.3389/fpsyg.2012.00533

**Published:** 2012-12-03

**Authors:** Meagan Yee, Susan S. Jones, Linda B. Smith

**Affiliations:** ^1^Department of Psychological and Brain Sciences, Indiana UniversityBloomington, IN, USA

**Keywords:** visual object recognition, shape bias, word learning, development, infants

## Abstract

Two of the most formidable skills that characterize human beings are language and our prowess in visual object recognition. They may also be developmentally intertwined. Two experiments, a large sample cross-sectional study and a smaller sample 6-month longitudinal study of 18- to 24-month-olds, tested a hypothesized developmental link between changes in visual object representation and noun learning. Previous findings in visual object recognition indicate that children’s ability to recognize common basic level categories from sparse structural shape representations of object shape emerges between the ages of 18 and 24 months, is related to noun vocabulary size, and is lacking in children with language delay. Other research shows in artificial noun learning tasks that during this same developmental period, young children systematically generalize object names by shape, that this shape bias predicts future noun learning, and is lacking in children with language delay. The two experiments examine the developmental relation between visual object recognition and the shape bias for the first time. The results show that developmental changes in visual object recognition systematically precede the emergence of the shape bias. The results suggest a developmental pathway in which early changes in visual object recognition that are themselves linked to category learning enable the discovery of higher-order regularities in category structure and thus the shape bias in novel noun learning tasks. The proposed developmental pathway has implications for understanding the role of specific experience in the development of both visual object recognition and the shape bias in early noun learning.

## Introduction

Language and visual object recognition are domains of human intelligence that impact almost all cognitive systems, and potentially also influence each other. Here we consider two developmental phenomena that imply a link between early object name learning and age related changes in the visual representation of object shape. Research on children’s learning of common nouns has emphasized the importance of attention to object shape as a predictor of rapid noun vocabulary growth (e.g., Poulin-Dubois et al., 1999; Smith et al., 2002; Gershkoff-Stowe and Smith, 2004). One commonly used experimental task measures children’s attention to shape over other perceptual dimensions when learning names for novel things (Landau et al., 1988). In this task, children are presented with a novel object, told its name (also novel) and then asked to indicate other things with the same name. Shape matches are put into competition with color, texture, or size matches. Young children consistently generalize the name to new instances by shape. This “shape bias,” becomes increasingly robust between 18 and 24 months of age and is more strongly related than is age to the number of nouns in individual children’s vocabularies (Gershkoff-Stowe and Smith, 2004). During this same developmental period, children also develop sparse representations of the three-dimensional shapes that characterize common basic level categories (Smith, 2009). The task used to measure this ability is known as “shape caricature recognition.” In this task, children are presented with abstract three-dimensional representations of common things – hats, chairs, cats – constructed from 2 to 4 geometric volumes so as to represent only the major object parts in their proper spatial relations (Smith, 2003). These representations provide only shape information with no other perceptual properties to support or compete with shape in object recognition. Between 18 and 24 months of age, young children become able (as are adults – Biederman, 1995) to recognize instances of common object categories given only these sparse shape representations. Shape caricature recognition, like the shape bias, is more strongly related to noun vocabulary size than age (Smith, 2003; Pereira and Smith, 2009).

These two tasks are both about children’s attention to object shape and are both related to vocabulary development, but they measure quite different abilities – one the ability to attend to shape over other properties, and the other the ability to recognize familiar categories from sparse shape information alone. These two abilities have never been studied in the same children. This is in part because the shape bias is usually conceptualized in terms of, and is part of the literature on, children’s knowledge about how different classes of words map to different kinds of meanings (e.g., Soja et al., 1991; Imai et al., 1994; Xu et al., 2009). Shape caricature recognition, in contrast, is about visual object recognition (Smith, 2009). Below, we provide the background on the two developmental trends, and then formulate two opposing hypotheses about their relationship.

**Figure 1:**
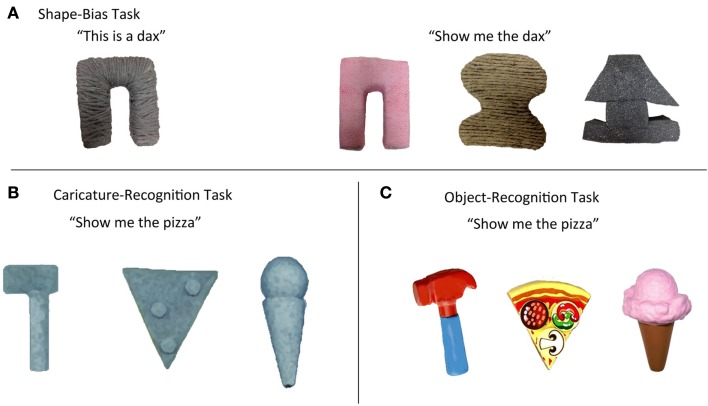
**Examples of stimulus sets used in the two experiments: (A) Shape bias – the named exemplar and three test objects matching in shape, surface texture, and color; (B) Caricature recognition – three sparse structural representations of the major parts of the characteristic shape of instances of common basic level categories; and (C) Object recognition – rich and typical toy representations of common basic level categories**.

The “shape bias” in children’s generalization of novel nouns to new instances was initially interesting because the bias suggested that very young children have expectations about the kinds of categories to which common nouns refer (Landau et al., 1988). There are a number of theoretical accounts that differ in the hypothesized nature of these expectations (e.g., Smith et al., 2002; Colunga and Smith, 2005; Kemp et al., 2007; Booth and Waxman, 2008). The present question about the shape bias does not concern these expectations about noun category mappings, but rather concerns the aspects of children’s representations of object shape that are measured by the task. In the standard version of the shape bias task, children are presented with novel made-up objects with very simple shapes, such as those shown in Figure 1A. The shape-matching test object is an *exact* shape match. The competing foils differ substantially from the target in shape with no overlapping parts or relational structure. Because the shapes are simple and the matches are exact, the task does not demand such advanced processes of visual shape representation as the parsing of the shape into component parts nor the analysis of the structural relations of those parts (Augustine et al., 2011). What the task does require is knowledge that shape and not color or size or texture is most likely the relevant dimension for determining category membership. This knowledge and the ability to selectively attend to shape in this task appear relevant to object name learning because 18- to 30-month-old children’s performances in the shape bias task are strongly related to their current noun vocabulary size (Smith, 1995; Gershkoff-Stowe and Smith, 2004; Perry and Samuelson, 2011; Hahn and Cantrell, 2012). More critically, the strength of the shape bias during this period also predicts children’s future vocabulary growth rate (Samuelson, 2002; Smith et al., 2002; Gershkoff-Stowe and Smith, 2004). Further relevant findings are these: the shape bias in artificial noun learning tasks is evident by 2 years of age (for review, see, Smith et al., 2010), but may be evident in some task contexts even earlier (e.g., Graham and Poulin-Dubois, 1999; Graham et al., 2004) and becomes substantially more robust with age (Smith et al., 2010; see also, Saalbach and Schalk, 2011); and the shape bias is correlated within individual children with the rate of productive vocabulary growth as well as vocabulary size, and measurably strengthens just before an increase in the rate of learning new nouns (Gershkoff-Stowe and Smith, 2004; Smith et al., 2010). The shape bias as measured in the experimental task of novel noun learning is delayed or lacking in children with atypical language development (Jones, 2003; Tek et al., 2008). Finally, prematurely teaching a shape bias to 17- to 19-month-olds leads to marked increases in their rates of outside-the-laboratory, that is, real-world, noun vocabulary growth (Samuelson, 2002; Smith et al., 2002; Perry et al., 2010).

Given the diagnostic relation between the development of a shape bias in novel noun generalization tasks and language development – and given that teaching a shape bias facilitates early vocabulary growth – it is critical to understand both the prior developments that support the emergence of the shape bias and the pathways through which biased attention to shape over other dimensions of similarity relates to object name learning. For example, one reason children’s performance in the shape bias task might predict future vocabulary development – despite the simplicity of the task and stimulus objects – might be that the task measures an early attentional bias that is prerequisite to building the visual object representations that support rapid category and word learning (Doumas and Hummel, 2010).

The study of shape caricature recognition was initially motivated by Biederman’s Recognition by Components (RBC) account of object recognition (1995). Biederman specifically proposed that humans form internal representations that are sparse geometric models, or caricatures, of the three-dimensional structure of object shape. These internally represented models were proposed to be built from a set of volumes called “geons” and were proposed to capture the whole object’s geometric structure independent of viewing perspective. These sparse structural representations of shape support category generalization by representing the individually unique instances of a category as having the same abstract shape; for example, kitchen chairs, dining chairs, and over-stuffed chairs are represented as having the same core geometric structure – seat and back. Several reviews have noted the lack of systematic study of the development of visual object recognition (Rentschler et al., 2004; Nishimura et al., 2009; Smith, 2009) and the likely protracted course of that development. The high-level visual processes that underlie these abstract caricatures of object shape appear to depend on category learning (e.g., Baker et al., 2002; Jiang et al., 2007; Doumas and Hummel, 2010) and thus might be expected to be related to early object category name learning in young children. Accordingly, several studies have examined whether young children, like adults, recognize instances of early learned noun categories given sparse geometric models of shape made from geon-like volumes (Smith, 2003; Pereira and Smith, 2009; Augustine et al., 2011). These studies compared recognition of richly detailed instances and three-dimensional “shape caricatures,” as shown in Figure 1B. Note that the rich instances can be recognized either by knowledge of category-specific surface features or by shape, but recognition of the shape caricatures requires knowledge of the abstract shape structure common to instances of the category. One study (Smith, 2003) examined 18- and 24-month-old children’s recognition using a non-linguistic play task. Play actions were scored as indicating recognition. For example, pretending to lick the ice cream or to hammer with the hammer was scored as indicating recognition, whereas banging, stacking, and rolling were not. In a name-comprehension task, children were shown three objects and asked to indicate one (e.g., “show me the pizza”). Both tasks yielded the same result: older children recognized the shape caricatures as well as they did the rich instances but younger children did not, recognizing the rich instances but performing at chance with the caricatures. These results provide two insights: first, representations of structural shape – of the kind posited by Biederman – are sufficient for object recognition in 2-year-olds, just as they are in adults. The fact that the older children in this sample – who are still very young – recognized the shape caricatures as well as they did richly detailed and typical toy examples shows that these children had abstracted the common geometric structure of the shapes of objects in familiar categories. Second, the fact that younger children recognized the detailed examples but failed to recognize the shape caricatures indicates a change between 18 and 24 months in the visual representations that support object recognition and category generalization. Additional evidence indicates that recognition of shape caricatures is more strongly correlated with productive vocabulary than with age (Smith, 2003; Pereira and Smith, 2009; Smith and Jones, 2011), that they support category generalizations of newly learned words (Son et al., 2008), and that the ability to recognize such sparse geometric representations is delayed in children with language delay (Jones and Smith, 2005).

Because shape caricature recognition requires the abstraction of the shape elements and relational structure common to the individually varied instances of a category, a reasonable hypothesis is that the development of shape caricature recognition follows and is perhaps dependent on attention to the specific exact shapes of individual things, the minimal visual skill that would seem necessary for success in the shape bias task. However, there is an alternative hypothesis derivable from one account of the origins of the shape bias. The attentional learning account (Smith et al., 2002; Yoshida and Smith, 2005; Perry et al., 2010) proposes that the shape bias in novel noun learning emerges through a multiple step process in which young children first learn the similarities that characterize individual basic level categories. This hypothesized first step supports the recognition of never-before-seen instances as members of known categories – for example, a novel tractor as a tractor or a novel chair as a chair. Then after some number of these categories are learned and because many things in basic level categories are similar in shape (Rosch, 1973; Samuelson and Smith, 1999; Colunga and Smith, 2005), children make the higher level generalization that is latent across basic level categories – that things in the same category are named by shape. Several different computational models – connectionist (Samuelson, 2002; Colunga and Smith, 2005), Bayesian (Kemp et al., 2007), and geometric (Hidaka and Smith, 2011) – have shown how a shape bias can emerge from this cross-category regularity. However, there is a major theoretical gap in the argument under all of these accounts that concerns the first step: how is it that young children represent object shape so as to recognize all varieties of tractors or chairs – with each individual instance having its own peculiar shape properties – as being abstractly and structurally the same kind of shape? By this line of reasoning, the development of sparse geometric representations of object shape – the kinds of representations measured by the shape caricature task – may be step one in the development of a generalized shape bias in novel noun learning. That is, the discovery of the latent structure across basic level categories – that things in the same basic level noun category have the same structural shape properties – may be dependent on first representing the abstract structural shape that characterizes known object categories.

The two experiments – the first a larger sample cross-sectional study and the second a smaller sample 6-month longitudinal study – examine for the first time the developmental ordering of success in the shape bias task and in the shape caricature recognition task within individual children. The experiments focus on the period between 18 and 24 months because past research shows increased performances in both tasks during this period. Because the standard versions of these experimental tasks have proved useful in predicting language learning and are diagnostic of language delay, we used standard versions of the two tasks while also trying to make them as similar as possible in terms of their response demands. In each task, the child was shown three potential choice items and the experimenter asked the child to indicate the named object. In the Shape Caricature task, the child was presented with three-dimensional sparse geometric representations of three different basic level categories as in Figure 1B and asked to indicate the one that the experimenter named. In the Shape Bias task, the child was first presented with a novel simple object, as shown in Figure 1A, told its name (also novel, e.g., “This is a dax”), and then presented with three choice objects. One of these objects was an exact shape match to the exemplar but differed in color and texture. The foils matched the exemplar in either color or texture but differed in shape. The child was asked to indicate which of the three choices was in the named category, “Show me the dax.” Finally, we also included an Object recognition task (see Figure 1C, using pictures in Experiment 1, and three-dimensional toys in Experiment 2) that should be easy for all children as the task requires children to map a common basic level noun to a rich and child-typical instance of the category. On each trial of this task, children were presented with three examples of different categories and asked to indicate the one named by the experimenter.

## Materials and Methods

### Participants

For the cross-sectional sample of Experiment 1, 55 children (27 males) aged 18–24 months with no known developmental disorders were recruited from a working and middle class population in a small Midwestern city. Three additional participants’ data were excluded from the analyses because of parents’ failure to comply with instructions (see below). For the 6-month longitudinal sample, 10 children (5 males; 5 females) none of whom participated in Experiment 1 were recruited from the same population. These children began the study at 18-months of age (*M* = 18.2, SD = 0.39) and were tested once every 3 weeks until they were 24-months old (*M* = 23.3, SD = 1.63) or until performance was greater than 80% on all three tasks. Thus, there was a maximum of nine test sessions per subject. Eight of the children participated in all nine test sessions, one participated in five sessions, and one in three sessions. All children were tested in the laboratory. The experimental procedures and recruitment of participants were approved by the Internal Review Board of Indiana University and informed consent was obtained from the parents of all participating children.

### Methods for experiment 1 (cross-sectional)

Each child participated in two practice trials to become familiar with the general procedure, and then in the three experimental tasks – a Shape Bias task, a Shape Caricature recognition task, and an Object recognition task. Stimuli for the practice trials were three-dimensional typical instances of common object categories – a flower, a spoon, and a duck – for which children in this age range normatively have receptive knowledge of the name (Fenson et al., 1993). The stimuli for the shape bias task consisted of two unique exemplars, one named “dax,” and one named “modi.” These were simply shaped objects that did not bear any obvious resemblance to real object categories. Two unique test sets – of three objects each – were made for each exemplar. Within each test set, one choice matched the exemplar exactly in shape but differed in color and texture, one matched in color but differed in shape and texture, and one matched in texture but differed in shape and color. One exemplar and test set is shown in Figure 1A. The mean volume of the objects was 345 cm^3^. The objects were constructed in the laboratory from wood, plastic, wire, hardened clay, or cloth. Each of the four unique test trials – two exemplars by two unique choice sets – was repeated twice to yield a test of eight trials. Eight unique random orders were used for testing, with roughly equal numbers of children assigned to each order.

The stimuli for the shape caricature task consisted of eight three-dimensional “geon-like” representations of eight basic level categories expected to be in the receptive vocabularies of most children in the age range (Fenson et al., 1993): pizza, brush, camera, ice cream, truck, hammer, cake, airplane. These Shape caricatures representations were constructed from 2 to 4 geometric volumes – cones, rectangles, pyramids, etc. – carved from Styrofoam, painted gray, and assembled to represent the major parts of the objects in their proper spatial relation – and averaged 935 cm^3^ in volume. Examples are shown in Figure 1B. Each of the eight categories was tested once. Three objects were used on each of the eight test trials: the target caricature and two caricatures drawn from the set of eight to serve as the foils. Eight unique random orders – with unique foil assignments for each target – were used for testing with roughly equal numbers of children assigned to each order.

The stimuli for the Object recognition task were eight color photographs of the typical instances of the objects on a white background, 12 × 17 cm in size, one each, of the eight categories tested in the shape caricature task (Figure 1C). The eight trials consisted of the target picture and two other pictures drawn from the set of eight to serve as the foils. Eight unique random orders – with unique foil assignments for each target – were used for testing with roughly equal numbers of children assigned to each order.

### Procedure

The child and experimenter sat opposite one another across a small table. The child sat either on the parent’s lap or in a chair beside the parent. Parents were instructed not to indicate in any way the choices – by word or by gesture – and a video camera directed at the parent recorded their behavior to ensure compliance (failure to comply to this request was responsible for the exclusion of three children’s data).

All tasks used a three-alternative-forced-choice procedure in which the child was verbally asked to select one object by name. In the Object and Shape Caricature Recognition tasks, the name was a basic level category noun. In the Shape Bias task, the name was the just-presented novel name of a novel exemplar object. The order of the Shape Caricature and Shape Bias tasks was counterbalanced across children. The Object Recognition task was always last. Within each task, the order of trials was randomly determined for each child, and within the Object and Shape Caricature task, the foils on each trial were randomly selected for each trial with the constraint that each unique picture or caricature was presented equally often as foils across trials and that no object (as target or foil) was repeated on two successive trials. The spatial location of the target – left, middle right – in all tasks was counterbalanced across trials within task.

On each of the two practice trials, the experimenter handed a common object – for example, a realistic silk flower – to the child and named it (e.g., “Look – here’s a flower!”). After a maximum of 20 s, the experimenter retrieved the object, placed it on the table near the experimenter but in full view of the child. The experimenter then placed another identical object (e.g., a replication of the flower) and the other two practice objects on a 25 cm × 50 cm tray with three compartments such that each choice object was in its own compartment. The child was then asked to get the target object by name (e.g., “Show me the flower here. Can you get a flower? Give me the flower”). Children were coached if necessary to manually select the object and given positive feedback. After two such practice trials, children proceeded to the main experiment. There was no coaching or rewarding feedback – just equanimity for all choices – in the main tasks.

The procedure for the eight Shape Bias trials was similar: the exemplar was named by the experimenter and examined by the child for a maximum of 20 s. Then with the exemplar still in view (but near the experimenter), the choice objects were put into the tray in randomly determined locations while the tray was beyond the child’s reach. Next, with the experimenter looking directly into the child’s eyes and not at the tray, the experimenter asked for the novel object and pushed the tray to within the child’s reach. The procedures for the Shape Caricature and Object Recognition tasks were nearly identical with one key exception: *there was no named exemplar object in those two tasks*. Instead, the three choice objects were put in the tray out of the child’s reach, the target object was named with the experimenter looking directly into the child’s eyes, and the tray was pushed to within the child’s reach. Thus the Shape Caricature Recognition and Object Recognition Tasks involved memory recognition from internal representations activated by the name rather than matching to an exemplar as in the Shape Bias task. The phrasing in all three tasks was held the same: “Show me the _____. Get the ______.” In all three tasks, the first object handed over by the child was recorded as the child’s choice. The correct choice in the Object Recognition task and in the Shape Caricature task was choosing the object representing the named category. The correct choice in the Shape Bias task was choosing the shape-matching object. These were coded from video by a blind coder; the experimenter also coded a randomly selected 25% of the choices by the children to check on reliability. Agreement was 97% as these responses are not ambiguous.

Following the experiment, the parent completed the MacArthur-Bates Communicative Development Inventory (CDI; Fenson et al., 1993), a widely used and reliable checklist measure of productive vocabulary that includes the first 600+ words normatively produced by children up to 30 months of age.

### Methods for experiment 2 (longitudinal)

All aspects of the procedure and design were the same as in Experiment 1 with the following exceptions. At the beginning of each test session, parents were asked to update a vocabulary checklist, reporting the words that their child produced using the Bates-MacArthur CDI as in Experiment 1. At each session, children participated in three tasks, using the same three-alternative forced-choice procedure for each task as described in Experiment 1. To increase the generality of the conclusions across the two studies (and because of what proved to be the structural fragility of some stimuli), the specific stimuli used in the tasks also included some new additions. For the Shape Caricature Recognition Task, the eight object categories were bird, key, spoon, boat, pizza, couch, shovel, hammer. Again, these three-dimensional shape caricatures were constructed from 2 to 4 three-dimensional geometric volumes (carved from Styrofoam and painted gray) in proper spatial arrangement and were the same size as those in Experiment 1. For the Object Recognition Task, more engaging lifelike toy representations rather than pictures were used. The toys were the same overall size as each other and the caricatures. The Shape bias task was the same as Experiment 1 but also used newly made but comparable exemplars and choice objects.

## Results

### Experiment 1: Cross-sectional sample

Because vocabulary rather than age has been the principle predictor of both the shape bias and shape caricature recognition (e.g., Pereira and Smith, 2009; Smith et al., 2010), the first set of analyses partitioned children into three Noun Vocabulary groups: low – 55 or fewer nouns; medium – 56–125 nouns; and high – more than 125 nouns. Table 1 provides the numbers, the average noun vocabulary and total vocabulary size, and the average age of children in each Vocabulary group.

**Table 1:** **Number of participants in the three vocabulary groups in Experiment 1, and the mean ages, noun vocabulary, and total vocabulary sizes for three groups**.

Vocabulary Group	Age	Nouns	Total Vocabulary
Low (fewer than 55 nouns), *n* = 20	*M* = 20.0 (18–24)	*M* = 25.9 (5–54)	*M* = 54.0 (7–120)
Medium (56–125 nouns), *n* = 18	*M* = 21.2 (19–24)	*M* = 89.7 (58–123)	*M* = 194.7 (103–346)
High (more than 126 nouns), *n* = 17	*M* = 22.65 (20–24)	*M* = 176.0 (126–300)	*M* = 398.5 (217–612)

*Ranges are given in parentheses*.

**Figure 2:**
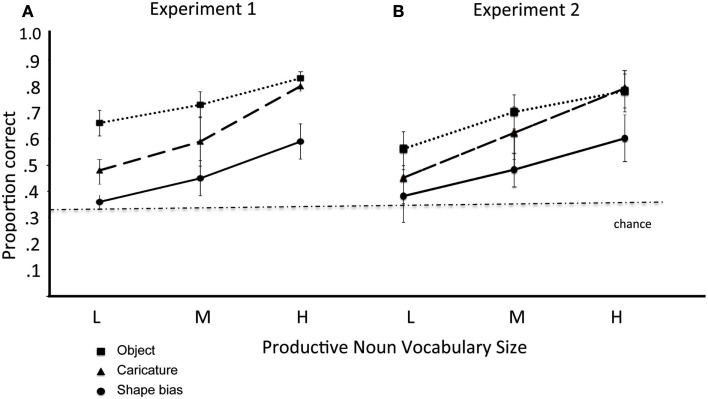
**(A)** For Experiment 1, and **(B)** for Experiment 2: mean proportion correct and standard errors in the Object recognition, Caricature recognition, and Shape bias tasks as a function of Vocabulary Size – Low (less than 55 nouns), Medium (56–125 nouns), and High (more than 125 nouns).

Figure 2A shows the mean scores of each group on the Shape Bias, Shape Caricature Recognition, and Object Recognition tasks. As is evident in the figure, performance in all three tasks increased as a function of vocabulary group, and children correctly selected the named target most often in the Object Recognition task. Critical to the main issue, however, is that children selected the correct test object more often in the Shape Caricature task than in the Shape Bias task in all three noun vocabulary groups. Scores on these three tasks were entered into a 3 (Noun Vocabulary Group) × 3 (Task: Shape Bias, Shape Caricature, Object Recognition) analysis of variance for a mixed design. The analysis yielded significant main effects for both Noun Vocabulary Group [*F*(2, 52) = 8.011, *p* = 0.001] and Task [*F*(2, 104) = 28.34, *p* < 0.001]. *Post hoc* analyses (*t*-tests with Bonferroni correction) indicated that children performed better on the Object Recognition task than in the Shape Caricature task [*t*(54) = 4.15] and performed better on the Shape Caricature task than on the Shape Bias task [*t*(54) = 4.32]. One-way ANOVA’s showed Vocabulary Group differences in Shape Bias scores [*F*(2, 52) = 4.65, *p* < 0.02] and Shape Caricature Recognition scores [*F*(2, 52) = 10.01, *p* < 0.001], but not in Object Recognition [*F*(2, 52) = 2.03, NS]. There was no interaction between Noun Vocabulary Group and Task [*F*(4, 104) = 0.78, NS]. Instead, average performance in the Shape Caricature task was better than average performance in the Shape Bias task by about the same margin in all three vocabulary groups.

Table 2 presents the full set of pairwise linear correlations among the variables in the study. With one exception, all the variables and measures were strongly correlated, a fact that does not implicate causal relations, but which does indicate a period of change in both object name learning and object recognition. In particular, children’s performances in the Shape Bias and Shape Caricature Recognition tasks were correlated and performance in both tasks were related to noun and total vocabulary size, as was recognition of the rich and detailed pictures in the Object Recognition task. However, whereas recognition of the Rich instances in the Object Recognition task was related to Shape Caricature recognition, it was not related to the strength of the Shape Bias, a fact that suggests that simply knowing the names of things is not sufficient for the development of the Shape Bias. We will return to this idea in the General Discussion. Vocabulary was a better predictor of performance in the Shape Bias and Shape Caricature Recognition tasks than was age. Further, when Shape Bias scores were regressed in a step-wise fashion on Age and Nouns, only Nouns were a significant predictor [*R*^2^ = 0.19, standard coefficient = 0.43: *F*(1, 53) = 12.06, *p* = 0.001]: and when Shape Caricature Recognition scores were regressed on the same two variables, again only Nouns significantly predicted the children’s scores [*R*^2^ = 0.31, standard coefficient = 0.56: *F*(1, 53) = 23.61, *p* < 0.001].

**Table 2:** **The pairwise linear correlations among the variables in Experiment 1**.

	Nouns	Total vocabulary	Object recognition	Caricature recognition	Shape bias
Age	**0.58**	**0.59**	**0.43**	**0.43**	**0.33**
Noun vocabulary		**0.97**	*0.32*	**0.56**	**0.43**
Total vocabulary			*0.32*	**0.56**	**0.43**
Object recognition				**0.62**	0.18
Caricature recognition					**0.41**

*Italics, *p* < 0.05; Bold *p* < 0.01*.

The pattern of performance in the Shape Bias and Shape Caricature tasks is consistent with the hypothesis that children, on average, develop structural shape representations of common categories *before* they develop a robust shape bias in generalizing names for novel objects. This result thus is consistent with the proposal that shape caricature development is part of the pathway leading to the development of a robust shape bias in novel noun generalization tasks. If this hypothesis is correct, individual children in this study should show a robust shape bias only if they already recognize basic level categories given caricatures of structural shape. Accordingly, we calculated the conditional probabilities in each noun group that a child would “pass” one task given that they had “passed” the other. Chance level performance on each task was 0.33, so passing for each task was defined as a proportion of 0.62 correct responses (at least five of eight correct responses, cumulative binomial probability by chance for each child = 0.084). By these criteria, the conditional probability that a child who passed the Shape Bias task also passed the Caricature task was consistently high across noun vocabulary groups (Low 0.80; Medium 0.70; High 0.92, binomial probability that this proportion of children would pass both <0.001 in all cases). That is, if a child in any vocabulary group showed a consistent shape bias in novel noun generalizations, it was highly likely that that child also recognized shape caricatures of common categories, a pattern consistent with the proposal that a robust shape bias depends on an already developed representation of the sparse geometric shapes of things in basic level categories. In contrast, the probability that a child who passed the Caricature task would also pass the Shape Bias task depended on vocabulary group (Low 0.33; Medium, 0.50; High 0.75, the binomial probability that these proportions of children would pass is significant only for the High Vocabulary group, *p* < 0.001). In sum, across vocabulary groups, success in Shape Caricature recognition does not predict success in the Shape Bias task and success on both tasks only characterizes the children with the largest noun vocabulary size. The overall pattern is consistent with the hypothesis that shape caricature recognition precedes the shape bias in novel noun generalization tasks, and with the proposal that the shape bias in novel noun generalizations depends, at least in part, on the recognition of the common structural shape of instances of known categories.

### Experiment 2: Longitudinal sample

Table 3 shows the means and ranges for the number of nouns and words in productive vocabulary and performance on the Object Recognition, Shape Caricature, and Shape Bias tasks. As is evident, this was a period of marked noun vocabulary growth for all children. However, as the ranges make clear, children entered the experiment with considerable differences in the sizes of their vocabularies and likewise ended the experiment with wide differences in vocabulary size. These individual differences in noun vocabulary growth are a well-known characteristic of this developmental period (Fenson et al., 1993).

**Table 3:** **The mean ages, noun vocabulary size, total vocabulary size, and mean performance in the three tasks for children at the first session and the last session for the 6-month longitudinal sample of Experiment 2**.

	Age	Nouns	Total vocabulary	Object recognition	Caricature recognition	Shape bias
First session	*M* = 18.2 (17.5–18.75)	*M* = 52.4 (14–176)	*M* = 96.3 (25–310)	*M* = 0.60 (0.50–1.00)	*M* = 0.53 (0.25–0.75)	*M* = 0.50 (0.17–0.83)
Last session	*M* = 23.3 (19.75–23.3)	*M* = 213.4 (150–301)	*M* = 426.3 (283–593)	*M* = 0.87 (0.50–1.00)	*M* = 0.83 (0.40–1.0)	*M* = 0.69 (0.33–1.0)

*Ranges are given in parentheses*.

As a first analysis of the relation between the development of the Shape Bias and Shape Caricature Recognition, we partitioned each child’s sessions into the same three Low, Medium and High Noun Vocabulary windows used in Experiment 1. Each child’s mean performance across sessions in each Vocabulary Size window was determined for the three tasks and submitted to an ANOVA for 3 (Vocab Size) × 3 (Task) repeated measures design; three subjects had starting vocabulary sizes larger than the upper boundary for the initial window and so for the missing data within those windows, the group mean was used. Figure 2B shows the mean performances in the three tasks by this vocabulary grouping. The pattern in the figure and the results of the ANOVA are highly similar to those of the cross-sectional sample in Experiment 1. There was a main effect of Vocabulary Size, *F*(2, 18) = 23.54, *p* < 0.001, with performance in all three tasks increasing as a function of the number of nouns in productive vocabulary, and a main effect of task, *F*(2, 18) = 28.28, *p* < 0.001. *Post hoc* analyses (*t*-tests with Bonferroni correction) indicate that children performed better in the Object Recognition than in the Shape Caricature task [*t*(9) = 3.60], and better in the Shape Caricature task than in the Shape Bias task [*t*(9) = 4.02]. The interaction did not approach significance, *F*(4, 36) < 1.00. Thus when analyzed in terms of the same vocabulary size groups, the pattern from the small sample longitudinal study is the same as from the larger sample cross-sectional study, increasing confidence that findings from these longitudinal data are generalizable to larger samples of children.

The key question for the longitudinal study is the temporal ordering of individual children’s success in the Shape Caricature and the Shape Bias task, because this temporal ordering provides information about causal relations between the two. Therefore, using the same criterion for success as in Experiment 1 (0.62 correct in a task), we defined the session at which Shape Caricature recognition and the Shape Bias “emerged” as the session at which a child first achieved that level of performance with the added stricture that the child’s performance never went below that level in a future session. We used the same criterion to measure the session in which children passed the Object Recognition (typical toy) task. All children at some point in the longitudinal sessions met the criterion for passing all tasks except three children: one never reached threshold in any of the three tasks, and two never achieved this level of success on the Shape Bias task. Children who did not reach the passing criterion in the nine sessions were given a “session” score of “10” for that task. This approach assumes that these children will eventually show a shape bias, an assumption that is warranted by the large literature showing robust shape biases in typically developing 3 year olds (see Smith et al., 2010 for review) and by the findings of the present cross-sectional study which show that, once a sufficient vocabulary size is reached, a robust shape bias is observed. However, if these children as expected achieve a shape bias it must be *after* their recognition of the shape caricatures. Thus the score of 10 underestimates the developmental timing of these skills for these children and is therefore the conservative statistical strategy. The mean session for passing the three tasks was 3.5, 4.8, and 6.2 for the Object Recognition, Shape Caricature, and Shape Bias tasks respectively and these differences are reliable [*F*(2, 18) = 36.47, *p* < 0.001]. *Post hoc* comparisons (*t*-tests with Bonferroni corrections) indicate a reliable difference between these measures for object recognition and the shape bias [*t*(9) = 3.62], with differences between object recognition and shape caricature recognition and between shape caricature recognition and the shape bias just missing conventional standards). This pattern tells us that the skills required to perform well in the shape caricature task do not depend on having the full complement of skills that enable children to perform well in the shape bias task. Thus, this pattern supports the conclusion that success in the Shape Caricature Recognition task precedes success in the Shape Bias task. Moreover, at the individual level there was only one child who showed a reversal and passed the Shape Bias task without having already passed the Shape Caricature task (and that child passed the shape caricature task in the very next session). Overall, this pattern supports the conclusion from Experiment 1 that the development of structural representations of the three-dimensional shapes of common things precedes and thus does not depend on the development of the skills reflected in the shape bias task.

**Figure 3:**
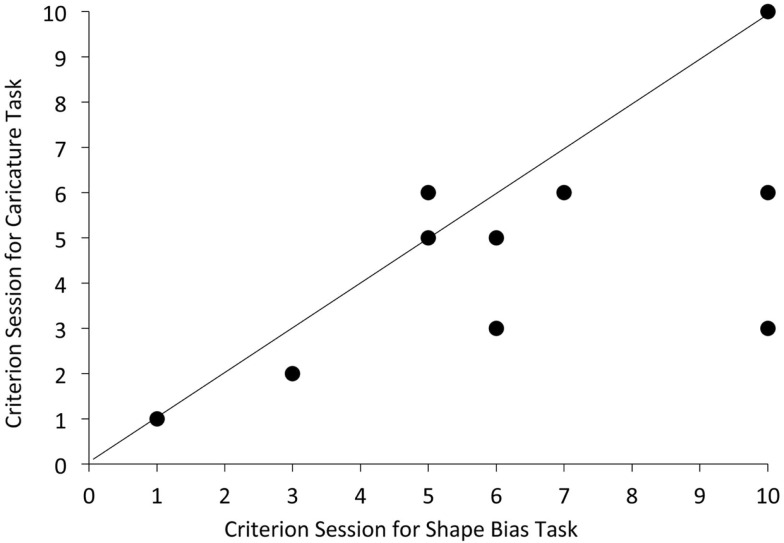
**Scatter plot of criterion sessions for success in the Caricature recognition task and Shape Bias task for the 10 children in the 6-month longitudinal sample in Experiment 2**.

Figure 3 shows the scatterplot of the sessions at which criterion performance was achieved in the shape bias task as a function of the session at which criterion performance was achieved in the caricature recognition task. If these two achievements were developmentally close then all participants should fall near the diagonal. If the Shape Bias emerged with some constant delay after Shape Caricature recognition, then the participants should fall near a line with a slope less than 1. Given the small sample, and the fact that three children did not succeed in the shape bias task before the study ended (a fact which supports the developmental priority of shape caricature recognition), it is not possible to measure the lag between success in the two tasks with any confidence. However, within this small sample, the timing of the two achievements is correlated (*r* = 0.61). Moreover, the pattern in Figure 3 makes clear that (1) success in the Shape Bias task lags behind Shape Caricature recognition and (2) the length of the lag varies considerably for individual children in this sample. Thus, while forming Shape Caricature representations for basic level categories might contribute to the development of the Shape bias, it appears not to be sufficient. The pattern in Figure 3 suggests that whatever the additional factors relevant for the development of the shape bias might be, they vary across children.

One might expect noun vocabulary size to be a good predictor of whether the shape bias emerges soon after or long after shape caricature recognition, since the emergence of both the shape bias and shape caricature recognition have been shown in previous research to be correlated with noun vocabulary size as well as in Experiment 1 (see Smith et al., 2010 for review). The noun vocabularies of these children as reported by their parents are markedly different at the point at which they meet criterion in the Shape Caricature recognition task, ranging from 50 to 210 nouns. However, there is a reliable correlation across the nine children who reached criterion on the Shape Caricature task (including the child who met criterion in session 1) between the vocabulary size at which they showed the Shape Bias and the gap in number of sessions between reaching criterion for the Shape Caricature task and the Shape Bias task [*r*(8) = 0.59, *p* = 0.05, mean gap in sessions 1.4, range −1 to 7). That is, in this small sample, children who had larger vocabularies when they first succeeded in the Shape Caricature task showed quicker subsequent development of the Shape Bias. This is a very small sample, and measured correlations are not stable across such small samples, but the observed relation suggests a new hypothesis that the development of a shape bias – generalizing newly learned object names to new instances by shape – depends on the representation of the abstract geometric shape that characterizes instances of early learned basic level categories *and* a sufficient number of already learned noun categories.

One limitation of this work is the use of the parent report of noun vocabulary size. Although the MCDI is a reliable and valid predictor of children’s *relative* language development in comparison to their peers and their future language development (e.g., Pan et al., 2004), it is likely to be a noisy measure of the actual words known by individual children (Styles and Plunkett, 2009; Mayor and Plunkett, 2011). As a further cautionary note, one of the participants did not reach criterion in any task and thus was given session criterion scores in each task of 10. That child’s Noun Vocabulary at the start was 20 nouns and was, by parent report, 205 nouns at the end, which places the child in the middle of the pack of 10 children; this child, however, never reached the five out of eight correct criterion even in the Object Recognition Task which is a receptive language task using typical and richly detailed objects. This fact raises questions about the parent-reported level of vocabulary development or the child’s behavioral compliance in the tasks. Further, although previous studies of shape caricature recognition and the shape bias in novel noun generalizations have used parent report of children’s productive noun vocabularies, the underlying assumption is not that the nouns the child says or even can comprehend are critical; rather vocabulary is used as a proxy measure of children’s knowledge of common object categories (e.g., Smith, 2003; Colunga and Smith, 2005). Standard measures of productive or receptive vocabulary may be imperfect indices of the relevant knowledge for shape caricature recognition or for the shape bias as total vocabulary measures do not, for example, tell us whether a child who produces or appears to comprehend a noun recognizes the full range of instances of the noun category or recognizes only some limited set of instances – or perhaps only a single instance, such as her own dog or her own toy bunny. Critically, it may be the extent and kind of knowledge of category instances across many categories that is the key to the formation of the over-hypothesis that “things in general are named by their shape.”

## Discussion

The present study was motivated by prior findings concerning the development of a shape bias in novel noun learning and the recognition of instances of basic level categories from sparse shape information. Past work showed that both developments occur between 18 and 24 months: both are related to early noun vocabulary size, and both are about object shape – but different aspects of attending to object shape. As a first step to understanding these developmental relations, the two experiments tested two hypotheses about the order of development of the two abilities in individual children. The results from both the cross-sectional and longitudinal samples support the hypothesis positing shape caricature recognition before the shape bias: children can recognize basic level categories from sparse shape information before they reliably extend an object name to objects that are identical in shape but differ in color and texture. These findings do not determine or identify the causal relation, if any, between the two developmental trends. However, they constrain potential hypotheses. There are four hypotheses about these causal relations in light of the results: (1) the two abilities are unrelated; (2) the shape bias – the ability to selectively attend to shape ignoring color and texture – contributes and is prerequisite to the development of shape caricature recognition; (3) shape caricature recognition is prerequisite to and supports the development of the shape bias; and (4) the two abilities co-develop, such that new partial knowledge in each fosters incremental development in the other. We consider each of these in turn.

The present results, which show a consistent developmental ordering as well as correlations between shape caricature recognition and shape bias scores, argue against the first hypothesis that the two achievements are unrelated. The extant literature, which shows in addition that both achievements are lacking in late talkers (Jones, 2003; Jones and Smith, 2005), and that both are correlated with vocabulary size (Smith, 2003; Smith et al., 2010) also strongly suggests some developmental relation. The present results also contradict the most straightforward prediction from the second hypothesis, that the shape bias is prerequisite to shape caricature recognition. As noted in the introduction, such a developmental ordering seemed mechanistically plausible in that children might have to preferentially attend to shape over other dimensions such as color and texture in order to discover the common geometric shape properties of members of the same basic level categories. This hypothesis is not strictly ruled out: as we discuss below, some components of a shape bias too weak to show in the present task might contribute to shape caricature recognition. However, the level of selective attention to shape needed to yield a shape bias in the standard novel noun generalization, task does not appear to be necessary to a robust ability to recognize basic level categories from sparse representations of object shape. Thus, the present evidence is more consistent with the final two hypotheses – that shape caricature recognition is prerequisite to the shape bias or that the two skills co-develop incrementally.

One might argue against the strong ordering hypothesis, that sparse structural representations of the shapes characteristic of basic level categories are *prerequisite* to the development of a robust shape bias in novel noun learning, by noting that the developmental ordering of performances in the two tasks will depend on the chosen measuring tasks. We attempted to make the two tasks as comparable as possible. Nonetheless, it is in principle possible to design a task measuring attention to shape over other properties in the context of object name learning that is easier than the present one, and thus in which children might succeed before they succeed in the present Shape Caricature Recognition task. The task we used to measure the shape bias is the most widely used version and the only one that has repeatedly been shown to be strongly related to noun vocabulary size and, more critically, to predict the *future* language development of individual children between the ages of 17 and 24 months (Samuelson, 2002; Smith et al., 2002; Gershkoff-Stowe and Smith, 2004). However, several studies using preferential looking measures or other methods in much younger infants found biased attention to shape in naming contexts that was not correlated with vocabulary size (Graham and Poulin-Dubois, 1999; Graham et al., 2004). This early “shape bias” could be due to different mechanisms (see, for example, Diesendruck and Bloom, 2003) or could be an early precursor of the later emerging shape bias in the standard noun generalization task. Those demonstrations of an early shape bias unrelated to vocabulary remind that developing abilities rarely emerge all or none, and that developmental pathways may be complex and not unidirectional. That is, a perhaps early bias to attend to object shape may exist independently of object name learning but be made stronger by learning names for things and this strengthening may benefit from the discovery of the common shapes of members of the same basic level category.

**Figure 4:**
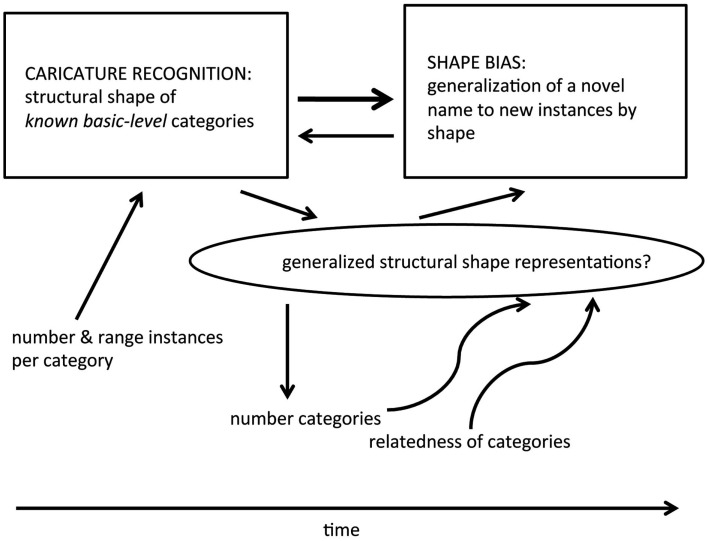
**Proposed developmental pathway and relevant experiences for the development of structural shape representations of common object categories and the shape bias in novel noun learning**. Caricature recognition for specific basic level categories depends on the number and range of experienced instances and precedes and supports the development of the shape bias. The generalized ability to represent the structural shape of even novel things may be an intervening skill that may depend on the number and kind of known basic level categories.

With this potentially complex and co-developmental process in mind, the remainder of the discussion is organized around the potential developmental pathways shown schematically in Figure 4. The figure shows (in the boxes) the two abilities – representation of the structural shapes that characterize known basic level categories, and biased generalization of a newly learned object name to new instances by shape. In line with the present results, shape caricature recognition in the figure precedes the shape bias. Also shown, in lower case text, are the hypothesized experiential factors that may be critical to the emergence of caricature representation and the shape bias: the number of instances known for any single category, the range of instances, and the number of categories. In the oval is a hypothesized additional ability that may be a critical intervening modulator with respect to the relations among shape caricature recognition, the shape bias, and the growth of object name vocabulary. As is the case in developmental process more generally (Blumberg, 2006; Stiles, 2008; Stiles and Jernigan, 2010; Sheya and Smith, 2010), the pathways may be complex, multi-causal, possibly bi-directional, and redundant such that there are multiple routes to the same end.

### The structural representation of three-dimensional shape

Mature human visual object recognition is fast, robust under degraded viewing conditions, and capable of recognizing novel, indeed unusual, instances of a very large number of common categories (Cooper et al., 1992; Pegna et al., 2004; Fize et al., 2005). For example, in their everyday lives, people routinely recognize the dog whose nose is sticking out from the blanket, the highly unique modernistic chair, and the cup on the table as a particular and favorite cup. An emerging consensus is that there is more than one route – and no single mechanism – underlying the full range of these abilities (Hummel, 2000; Hayward, 2003; Peissig and Tarr, 2007; Smith, 2009). Because there are multiple routes to object recognition, the recognition of known basic level categories *does not require* the representation of or even attention to overall shape, but may be accomplished on the basis of piecemeal diagnostic local features (Ullman et al., 2002; Pereira and Smith, 2009). For example, if something has a dog nose, it is likely to be a dog; if it has a bumper and wheels, it is likely to be a car. However, the evidence indicates that given experience with categories, perceivers also build more configural forms of representation (see Peissig and Tarr, 2007), and in the case of common (non-face objects) build configural representations in terms of the relational structure of the major geometric parts (Kourtzi and Connor, 2011). These representations, like the caricatures used in the present studies, may capture the sparse three-dimensional structure of object shape (Biederman, 1995). The developmental evidence suggests that, in general, infants begin with piecemeal feature representations and only slowly build structural representations of the whole (Pereira and Smith, 2009; Augustine et al., 2011; Jüttner et al., 2012). Thus, infants’ early recognition of instances of well-known categories may not be primarily based on the relational structure of the major shape components. From this perspective, shape caricature recognition is not a critical achievement because it is necessary for recognizing category instances but because these structural shape representations have advantages over local diagnostic features in supporting category generalization (Son et al., 2008), in deciding about actions on objects (Eloka and Franz, 2010), and in recognizing objects from multiple viewpoints (Jüttner et al., 2012). The present results and the pathway model in Figure 4, propose further that these representations may contribute to the development of the shape bias in novel noun learning and thus, at least indirectly, to the learning of names for things in basic level categories.

Although computational models (e.g., Doumas and Hummel, 2010) and some empirical findings (Augustine et al., 2011) suggest that category learning is critical to the emergence of structural shape representations, little is known about the precise nature of the relevant experiences for children or about the nature of the developmental trajectory. It seems likely that the formation of these representations requires viewing three-dimensional objects from multiple viewpoints so that those viewpoints may be dynamically integrated into a unified representation (Graf, 2006; Farivar, 2009). Several recent studies suggest that manual exploration may be a key factor in these developments (Soska et al., 2007; Pereira et al., 2010). Structural shape representations may also require experience with multiple category instances – easy chairs and rocking chairs – to pull out the relevant parts and relations. Finally, the ability to rapidly represent the structural properties of any complex shape – including the shapes of novel things (an ability not tested here) – may depend on experience with many categories and not just a few categories. This hypothesized more general representational skill – one that goes beyond well-known categories and is indicated in the oval in Figure 4 – may not be fully mature until quite late (Abecassis et al., 2001; Mash, 2006).

One early study suggested that young children could recognize the sparse shapes of even novel objects as well as the shape caricatures of known things (Smith, 2003). However, that study did not use foils that seriously challenged the quality or kind of representations underlying children’s choices, and they could have responded correctly merely by matching one component part of the richer real-life exemplar to the caricature. More challenging empirical examination of emerging skills suggests that children first form these representations for well-known categories and only subsequently are able to abstract the structural shape of novel things (Mash, 2006; Augustine et al., 2011). Two components of these caricature representations are (1) determining the global parts and (2) representing the spatial relations among those parts. Recent evidence suggests that the limiting ability in young children’s representation is the relational structure among parts. Young children show particular difficulty in discriminating both known and novel objects with the same parts in different relational organizations but they are generally well able to discriminate objects that differ only in a component part (Augustine et al., 2011). A further open question is whether (and if so how) the representation of the relational structure of objects depends on experiences within and across categories.

A better understanding of the development of structural shape representations appears critical to understanding the developmental relation between shape caricature recognition and the shape bias. If a generalized ability to represent three-dimensional shape depends on representing the relational structure of objects and if these generalized representations are key to developing a shape bias – or in using a shape bias to learn real-world categories – then we need to understand the experiences that support these developments.

### The shape bias in novel noun generalizations

There are both extensive empirical findings and multiple theoretical accounts of the shape bias in children’s early noun generalizations (e.g., Booth and Waxman, 2008; Colunga and Smith, 2008). Three classes of formal computational models have specifically considered the factors relevant to developmental changes in the shape bias and the relation of those changes to noun vocabulary growth. One formal account, a Bayesian model of word-category learning as hypothesis testing (Kemp et al., 2007), sought to understand how an over-hypothesis that things are named by shape could be confirmed on relatively *little evidence*, with the underlying assumption being that the shape bias forms when children have relatively small vocabularies and when many of those categories are at best imperfectly and not solely organized by shape (see also Yoshida and Smith, 2003). The two alternative computational theories – connectionist models (Colunga and Smith, 2005) and the geometric model (Hidaka and Smith, 2011) – assume children’s experiences provide dense, albeit individually imperfect, evidence, and assume that some critical mass of instances and categories is essential to forming the generalization about the relevance of shape to basic level noun categories. By these analyses, broader statistical evidence is needed because, again, although shape is generally important across many basic level nouns, it is not the only perceptual property relevant for many individual categories and it is not critical at all for some common categories (Samuelson and Smith, 1999; Yoshida and Smith, 2003; Perry and Samuelson, 2011). Thus rich and extensive experience with categories and their instances underlies the emergence of the shape bias in these accounts. Two training studies (Samuelson, 2002; Smith et al., 2002) have shown that training with a few categories is sufficient to yield a shape bias in children who do not yet show one (consistent with the Bayesian conceptualization of the learning problem). However, one recent study shows that the development of a shape bias in these children is more robust when training involves a wide variety of instances of individual categories (Perry et al., 2010), a result potentially consistent with the view that dense data on the structure of individual categories is a relevant factor.

The geometric analysis of basic level category structure offered by Hidaka and Smith (2011), an analysis conceptually related to the mathematical properties of self-organizing maps, provides a possible insight into why the range of exemplars matters. Individual instances are defined within a high-dimension feature space. The model shows how competition at the boundaries of near (that is perceptually similar) categories within this high-dimension space are critical to finding the much smaller set of dimensions that may be used to define the regions corresponding to specific basic level categories. An extended range that includes unusual rather than the most typical instances provides fodder for border-competition. If this mathematical analysis captures aspects of the relevant psychological processes then it would also imply that it is not just the number of categories and range of instances within a category that matter to the development of a shape bias but also the proximity of the categories in perceptual feature space. That is, by implication, knowing the categories BED, CHAIR, and TABLE or the categories TRUCK, CAR, and BUS might facilitate the development of a shape bias (and/or perhaps shape caricature representations) better than knowing BED, CAR, and DOG, because it is the *close* discriminations that may drive new low-dimensional forms of representation. This is a particularly interesting proposal in light of new evidence concerning children who are slow in their vocabulary development. Several new analyses suggest that the vocabularies of these children who are at risk for later language learning and processing difficulties are not simply small but sparsely structured so that the likelihood of knowing semantically related words is less than that of typically developing children and less than would be expected from a random learner (Beckage et al., 2011). If semantically related words refer to near categories in perceptual similarity space, then a limiting factor in the development of the shape bias may be the perceptual and conceptual relatedness of the basic level categories known by the child.

## Conclusion

Two of the most formidable skills that characterize human beings are language and our prowess in visual object recognition. Young children build these skills together which raises the possibility of mutual developmental dependencies. One domain in which these interactions may be particularly important is in the visual representation of shape and in the early learning of object names. Things in the same basic level noun categories are typically similar in shape (Rosch, 1973), and young children when learning new object names become biased to extend names to instances similar in shape (Landau et al., 1988). But shape itself is a visual enigma, describable in multiples ways (Graf, 2006), and potentially represented in the nervous system in multiple ways. Learning object names, and the ranges of things that are in common noun categories, may teach one of those forms of shape representations. Finally, these sparse representations of structural shape also may be an important ingredient in children’s developing expectations of the kinds of things to which common nouns refer.

## Conflict of Interest Statement

The authors declare that the research was conducted in the absence of any commercial or financial relationships that could be construed as a potential conflict of interest.
